# Advancing Tailored Treatments: A Predictive Nomogram, Based on Ultrasound and Laboratory Data, for Assessing Nodal Involvement in Endometrial Cancer Patients

**DOI:** 10.3390/jcm13020496

**Published:** 2024-01-16

**Authors:** Ida Pino, Elisa Gozzini, Davide Radice, Sara Boveri, Anna Daniela Iacobone, Ailyn Mariela Vidal Urbinati, Francesco Multinu, Giuseppe Gullo, Gaspare Cucinella, Dorella Franchi

**Affiliations:** 1 Preventive Gynecology Unit, European Institute of Oncology IRCCS, 20141 Milan, Italy; annadaniela.iacobone@ieo.it (A.D.I.); ailyn.vidalurbinati@ieo.it (A.M.V.U.); dorella.franchi@ieo.it (D.F.); 2 Department of Clinical and Experimental Sciences, University of Brescia, 25123 Brescia, Italy; e.gozzini003@unibs.it; 3 Division of Epidemiology and Biostatistics, IEO European Institute of Oncology IRCCS, 20141 Milan, Italy; davide.radice@ieo.it; 4 Laboratory of Biostatistics and Data Management, Scientific Directorate, IRCCS Policlinico San Donato, San Donato Milanese, 20097 Milan, Italy; sara.boveri@grupposandonato.it; 5 Department of Biomedical Sciences, University of Sassari, 07100 Sassari, Italy; 6 Department of Gynecologic Surgery, IRCCS European Institute of Oncology, 20141 Milan, Italy; francesco.multinu@ieo.it; 7 Department of Obstetrics and Gynecology, Villa Sofia Cervello Hospital, University of Palermo, 90146 Palermo, Italy; gullogiuseppe@libero.it (G.G.); gaspare.cucinella@unipa.it (G.C.)

**Keywords:** endometrial cancer, lymph node metastasis, predictive nomogram, preoperative assessment, sentinel lymph node biopsy

## Abstract

Assessing lymph node metastasis is crucial in determining the optimal therapeutic approach for endometrial cancer (EC). Considering the impact of lymphadenectomy, there is an urgent need for a cost-effective and easily applicable method to evaluate the risk of lymph node metastasis in cases of sentinel lymph node (SLN) biopsy failure. This retrospective monocentric study enrolled EC patients, who underwent surgical staging with nodal assessment. Data concerning demographic, clinicopathological, ultrasound, and surgical characteristics were collected from medical records. Ultrasound examinations were conducted in accordance with the IETA statement. We identified 425 patients, and, after applying exclusion criteria, the analysis included 313 women. Parameters incorporated into the nomogram were selected via univariate and multivariable analyses, including platelet count, myometrial infiltration, minimal tumor-free margin, and CA 125. The nomogram exhibited good accuracy in predicting lymph node involvement, with an AUC of 0.88. Using a cutoff of 10% likelihood of nodal involvement, the nomogram displayed a low false-negative rate of 0.04 (95% CI 0.00–0.19) in the training set. The adaptability of this straightforward model renders it suitable for implementation across diverse clinical settings, aiding gynecological oncologists in preoperative patient evaluations and facilitating the design of personalized treatments. However, external validation is mandatory for confirming diagnostic accuracy.

## 1. Introduction

Endometrial cancer (EC) is one of the most common gynecological malignancies, mainly in developed countries [1]. The cornerstone of treatment for early-stage neoplasms involves total hysterectomy, bilateral adnexectomy and lymphadenectomy, where the assessment of lymph node status significantly influence the determination of recurrence risk and subsequent adjuvant treatment planning [2]. Nevertheless, lymphadenectomy is not recommended for low-risk cases, due to the elevated risk of complications and the absence of survival benefits [3,4,5,6]. Increasing evidence suggests that employing sentinel lymph node biopsy with pathologic ultra-staging in low- and intermediate-risk endometrial cancer results in a high detection rate (DR), sensitivity, and negative predictive value (NPV). Crucially, this approach does not negatively impact patients’ progression-free survival and overall survival while maintaining low operative complications [7,8,9,10]. Furthermore, when performed by skilled surgeons, sentinel lymph node biopsy demonstrates elevated sensitivity and NPV, even in high-risk and high-grade EC cases [11]. With increased surgeon proficiency, the technique has achieved a 5% false-negative rate, and most of the studies in the literature report a bilateral detection rate exceeding 85% [8,12,13,14,15]. Moreover, a recent retrospective analysis involving 1044 patients, by Mueller et al., showed the absence of nodal metastases in 510 patients with International Federation of Gynecology and Obstetrics (FIGO 2009) stage 1A, grade 1, or 2 [16]. In cases where sentinel lymph node mapping fails, the surgical protocol advises systematic pelvic side-specific lymphadenectomy. This is a measure aimed to address the potential threat of hidden nodal metastasis, but may lead to overtreatment [17]. Additionally, sentinel-node mapping is not available in all centers. In this scenario, the advent of the Proactive Molecular Risk Classifier for Endometrial Cancer (ProMisE) has profoundly transformed our understanding of this neoplasm, particularly in terms of prognosis and new therapeutic targets [18]. The molecular profile inherently constitutes an independent risk factor for lymph node metastasis at the time of diagnosis, as highlighted by Jamieson et al. [19].

Predictive models for preoperative risk assessment can be used in women diagnosed with EC to evaluate the risk of lymph node metastases prior to surgery. This helps prevent both undertreatment and overtreatment through unnecessary lymphadenectomies. Most published nomograms predicting lymph node metastasis utilize histological parameters and magnetic resonance imaging (MRI) to assess disease spread and identify suspicious lymphadenopathies [20,21,22]. Nonetheless, there is only moderate agreement in tumor grade between the endometrial biopsy and the hysterectomy specimen [23,24]. In addition to its crucial diagnostic role, transvaginal ultrasound (TV-US) is increasingly recognized as a valuable tool in the preoperative assessment of patients with EC. Indeed, it is now considered as an integral part of the pre-treatment workup for endometrial neoplasms in the ESGO/ESTRO/ESP guidelines [2]. TV-US, when performed by expert practitioners following standardized methodology, shows a predictive accuracy for local extension of endometrial cancer comparable to that of MRI. However, ultrasound holds advantage over MRI in terms of accessibility, patient tolerance, cost-effectiveness and time efficiency [25,26]. Eriksson et al. conducted a prospective study, relying on endometrial biopsy results as well as clinical and ultrasound characteristics. Despite the good diagnostic accuracy achieved by the scoring system, the formula for determining the percentage probability of lymph node positivity appears more complex to use without computer software, compared to the utilization of a paper nomogram [27].

To date, there are no specific tumor markers for the differential diagnosis of EC. The CA 125 assay is highly useful in ovarian cancer for both diagnostic purposes and treatment response monitoring. In contrast, it is not included in the standard diagnostic work-up of EC, due to its inability to offer differential diagnostic information. However, numerous studies have indicated that a preoperative CA 125 level > 35 IU/mL represents an independent risk factor for nodal positivity [28,29,30,31,32,33,34]. Additionally, growing evidence suggests that systemic inflammatory responders, such as the neutrophil/lymphocyte ratio, platelet/lymphocyte ratio, C-reactive protein, platelets, and fibrinogen, may play a crucial role in the development and progression of solid tumors. Specifically for endometrial cancer, studies have correlated a high neutrophil/lymphocyte ratio and elevated platelets with a higher likelihood of nodal metastasis [35,36]. The main objective of this study is to develop a preoperative scoring system using clinical, laboratory, and ultrasound parameters to predict the likelihood of lymph node positivity in patients undergoing surgical treatment for EC.

## 2. Materials and Methods

This retrospective monocentric study was conducted at the European Institute of Oncology (IEO), IRCCS, Milan, Italy. From April 2019 to June 2023, all consecutive patients diagnosed with EC who underwent surgical staging with nodal assessment were retrospectively included in the analysis. All enrolled patients provided consent for the data collection for research purposes, and the study was approved by the Institutional Review Board protocol code IEO UID (2418).

Data concerning demographic, clinicopathological, imaging and surgical characteristics were collected from the electronic medical records. The inclusion criteria were as follows: age ≥ 18 years old; histological diagnosis of endometrioid EC; performance of hysterectomy with salpingo-oophorectomy and nodal mapping (systematic lymphadenectomy or SLN); conventional pathological evaluation; molecular analyses; blood chemistry and TV-US performed by experienced practitioner, following the IETA consensus statement and at least 30 days before surgery [37,38]. Exclusion criteria included the following: consent withdrawal; missing preoperative variables; absence of nodal surgical mapping; advanced stage (FIGO 2009 stage IIIa and b-IV) at diagnosis; histological diagnosis of non-endometrioid EC; neoadjuvant chemotherapy; previous fertility sparing treatment; hematologic diseases. Ultrasound examinations were conducted with the patient in the lithotomy position and an empty bladder, adhering to standardized techniques outlined in the IETA consensus statement [38]. The examination entailed scanning the uterus from cornu to cornu in the sagittal plane and from the cervix to the fundus in the transverse plane. Measurements of the anteroposterior (AP) diameter of the uterus and endometrial thickness or, in the presence of an endometrial lesion, tumor thickness, were obtained in the sagittal plane. The minimal tumor-free margin (MFM) was measured in any plane where the distance from the tumor to the serosa appeared to be the smallest.

Subjective assessments of deep myometrial invasion (MI) ≥ 50% or cervical stromal invasion (CSI) were conducted on magnified images of the uterine corpus and cervix uteri, respectively. The presence of deep myometrial invasion was evaluated also, using the ratio between the AP diameter of the tumor and the AP diameter of the uterus at the point where the deepest myometrial invasion was identified (d/D). Cervical stromal invasion was evaluated also, by measuring the distance between the caudal margin of the neoplasm and the external uterine orifice.

Additionally, extrauterine spread was evaluated, including invasion of the serosa of the uterine corpus, parametrial invasion, invasion of the adnexa, presence of enlarged pelvic lymph nodes, and engagement of the bladder or rectal wall/mucosa, as well as assessments for carcinosis, ascites, and spread beyond the pelvic region.

This evaluation used transvaginal ultrasound, with or without additional transabdominal ultrasound, as deemed necessary by the examiner. Color/power Doppler examinations were conducted at a pulse repetition frequency of 0.3–0.9 kHz, with adjustments made to gain and pulse-repetition frequency, to ensure clear vessel definition. The standard approach for surgical staging involved minimally invasive techniques, predominantly laparoscopic or robotic-assisted type A hysterectomy with bilateral salpingo-oophorectomy and nodal surgical mapping. Nodal staging encompassed systematic pelvic with/without para-aortic lymphadenectomy and/or SLN biopsy. In case of SLN failure, lymphadenectomy of the non-detected hemi-pelvis was performed, in accordance with international guidelines [17]. Enlarged lymph nodes were excised, regardless of SLN mapping. Para-aortic lymphadenectomy was omitted unless sentinel or suspicious nodes were identified in the para-aortic region. Patients with measurable lymphadenopathy on preoperative imaging, evaluated according to RECIST 1.1 criteria, and not confirmed intraoperatively as bulky lymphadenopathy, underwent either systematic pelvic lymphadenectomy or systematic pelvic and para-aortic lymphadenectomy [39,40]. The surgical team consisted of specialized gynecological oncologists and fellows undergoing training in gynecologic oncology. SLN mapping was executed using cervical injection of indocyanine green (ICG), following the Memorial Sloan Kettering Cancer Center SLN algorithm [41]. As per this algorithm, SLNs were not subjected to intraoperative frozen-section analysis, but ultrastaging was consistently performed [42]. All histological specimens were evaluated by a dedicated gynecological pathologist. Lymph node metastases were categorized according to TNM8 into macrometastases (tumor clusters > 2 mm) and low-volume metastases, including micrometastases (tumor clusters 0.2–2 mm) and isolated tumor cells (ITCs) (single tumor cells or tumor clusters ≤ 0.2 mm) [43]. Patients were staged according to the 2009 FIGO staging system. Histological classification and the degree of glandular differentiation were classified according to the World Health Organization (WHO) and FIGO classification systems, respectively [2,44]. Molecular analysis of tumor tissue was performed on DNA extracted from formalin-fixed paraffin-embedded (FFPE) tumor tissue, using Promega Maxwell^®^ CSC DNA FFPE kit (CE-IVD) on Promega Maxwell^®^ CSC Instrument platform. The mutational status of the POL-E and TP53 genes were evaluated using next-generation sequencing (NGS) techniques within a panel comprising 26 tumor-related genes (Custom IEO). The molecular data were supplemented by immunohistochemical expression analysis of the p53 protein. Microsatellite status evaluation was performed using the Idylla^TM^ MSI Assay at 7 loci. The molecular data were supplemented by evaluation of immunohistochemical expression of MLH1, PMS2, MSH2, and MSH6. In instances of loss of MLH1 expression, hypermethylation of the MLH1 gene promoter was examined [45]. The ProMisE classification was applied to categorize patients into the four molecular classes: POLE-mutated (POLE), MMR-deficient (MMR-d), p53 abnormal (p53abn), or non-specific molecular profile (NSMP) [18,46].

Patients’ characteristics were summarized either by count and percent (categorical variables) or median and interquartile range (continuous variables), based on the significance of the Shapiro–Wilk normality test, and tabulated according to the nodal involve-mate. To build a predictive nomogram, a split-sample validation process has been used. Seventy percent of the whole data set was randomly allocated to a training cohort, to esti-mate the best logistic model parameters, and the remaining thirty percent was used for the validation process. Results of the univariate and multivariable logistic regression analyses are tabulated as odds ratios (ORs) and the receiver operating characteristic (ROC) area under the curve (AUC), alongside 95% confidence intervals (95% CI). Variables entering the multivariable stepwise regression model were pre-selected, considering their clinical relevance and their significance in the univariate test. Goodness of fit of the final model was tested using the Hosmer–Lemeshow test, as well as with the multivariable ROC AUC. Nodal involvement-predicted probabilities were plotted against ordered ranked patient ID for the training and validation cohorts. False-positive and false-negative rates were estimated by setting a value of 10% for the predicted probability, as a threshold. Between-group comparisons were carried out using either the Fisher’s exact test or the two-sample Wilcoxon test for categorical and continuous variables, respectively. All tests were two-tailed and considered significant at the 5% level. All analyses were carried out using SAS 9.4 (Cary, NC, USA) and Stata 18 (StataCorp. 2023. Stata Statistical Software: Release 18. College Station, TX, USA: StataCorp LLC).

## 3. Results

We identified 425 patients who underwent surgery for endometrioid EC from April 2019 to June 2023. After applying the exclusion criteria, the final analysis included 313 women.

The initial diagnosis was performed using different sampling methods. Predominantly, preoperative biopsy was performed through hysteroscopy (65.6%), while the remaining diagnoses were obtained using dilatation and curettage (4.1%), Pipelle (17.2%), or VABRA (3.2%). In 9 cases (2.9%), the specimen was spontaneously expelled from the endometrial cavity, while in 20 cases (6.4%), the method was not documented.

Minimally invasive surgery was the prevalent approach. Surgical staging was conducted via laparotomy in 58 out of 313 cases (18.5%), laparoscopy in 19 cases out of 313 (6.1%), and robot-assisted laparoscopy in 236 cases out of 313 (75.4%), using the Da Vinci Xi robotic platform (ab medica s.p.a., Cerro Maggiore (MI)).

Lymph node status was assessed in 277 out of 313 (88.5%) patients via SLN biopsy, in 16 out of 313 (5.1%) patients through systematic pelvic lymphadenectomy, and in 20 out of 313 (6.4%) patients through systematic pelvic and para-aortic lymphadenectomy.

In only 4 out of 277 (1.4%) patients, SLN biopsy failed, leading to the performance of systematic pelvic lymphadenectomy on the non-retrieval side, in accordance with guidelines.

At the histopathological examination of the lymph nodes, 52 out of 313 (16.6%) patients presented positive lymph nodes, including 39 cases with micro/macro metastasis, and 13 with isolated tumor cells.

### 3.1. Univariate Analysis and Multivariable Analyses

The study group was divided into a training cohort (n = 210 patients) and a validation set (n = 103 patients), through a random selection process.

Within the 210 patients of the training set, 126 (60%) were diagnosed with stage Ia EC, and 109/208 (52%) were categorized as low risk, according to the ESGO/ESTRO/ESP classification. Two patients had insufficient sample for molecular investigations. Among the remaining 208 patients, the distribution was as follows: NSMP molecular class in 91/208 cases (43.7%), MSI/MMR-d in 77/208 cases (37%), p53 mutation in 17/208 cases (8.2%), and POLE mutation in 23/208 cases (11.1%).

The clinical, demographic, laboratory, and ultrasound characteristics of patients in the training group are summarized in Table 1 for continuous variables and Table 2 for categorical variables.

Table 3 outlines variables that proved to be more objective, clinically relevant, and/or statistically significant in the univariate analysis of the training cohort.

No statistically significant differences were found in clinical or demographic characteristics between patients with and without positive lymph nodes at surgical staging. However, several laboratory parameters and ultrasound variables were statistically significant in the univariate analysis.

Among the laboratory parameters, in addition to CA 125 (*p*-value < 0.001), systemic inflammatory responders such as neutrophil and platelet counts showed significance, with a *p*-value of 0.01.

Significant ultrasound characteristics included tumor size indicators (all lesion diameters and endometrial thickness, *p* value < 0.001) and markers of disease extension in terms of myometrial infiltration (MFM and d/D, *p* value < 0.001), and cervical stromal involvement (distance between tumor and external os, *p* value < 0.001).

The stepwise multivariable logistic regression applied to the training-cohort-identified variables for the final model. The selected risk factors were the following: d/D expressed as a categorical variable (<50%–≥50%) with an odds ratio (OR) of 3.44 (95% CI 0.61–19.3, *p*-value 0.001), CA 125 expressed as a categorical variable (<35 U/mL vs. >35 U/mL) with an OR of 7.74 (95% CI 1.86–32.2, *p*-value < 0.001), MFM with an OR of 0.73 (95% CI 0.53–1.01, *p*-value 0.05), and platelet count with an OR of 1.01 (95% CI 1.00–1.02, *p*-value 0.13) (Table 4).

### 3.2. The Nomogram

**Figure 1:**
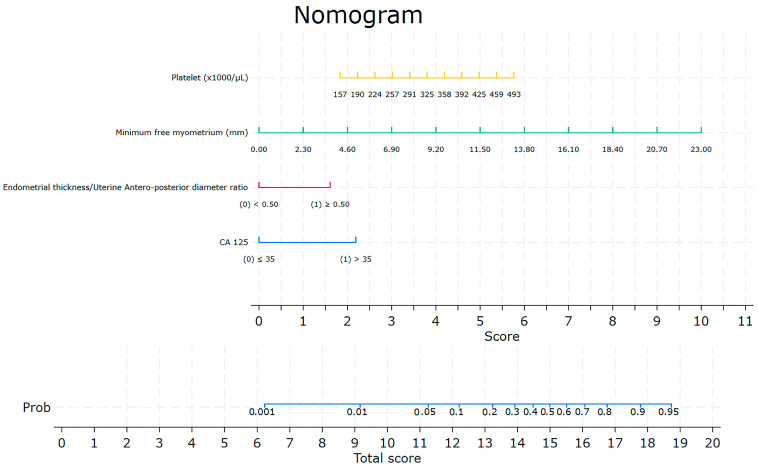
Nomogram for the prediction of nodal involvement.

The results facilitated the creation of a nomogram to predict the likelihood of lymph node positivity in surgically treated patients with endometrioid EC (Figure 1).

The parameters selected via univariate and multivariate analysis included platelet count (1000/microL–10^9^/mL), myometrial infiltration (<0.50–≥0.50), MFM (mm), and CA 125 (<35 vs. >35 U/mL).

**Figure 2:**
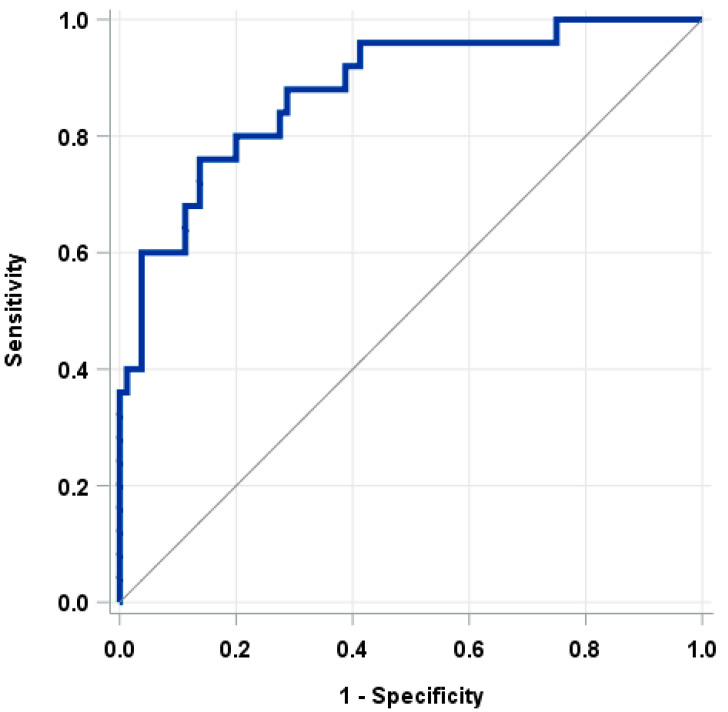
Diagnostic accuracy for the proposed model, AUC = 0.88.

The nomogram demonstrated high accuracy in predicting lymph node involvement, as indicated by an AUC of 0.88 in the ROC curve (Figure 2).

**Figure 3:**
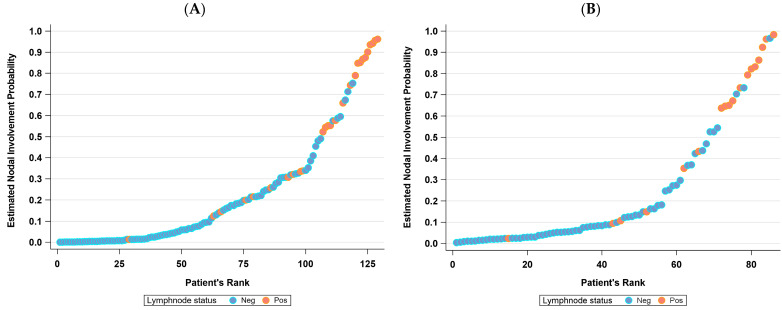
Lymph node involvement predicted probability vs. actual nodal status for the training (**A**) and validation (**B**) cohorts.

As illustrated in Figure 3, the predicted probability of lymph node involvement versus the actual nodal status remained consistent between the training and validation cohorts.

At the estimated cutoff of a 10% likelihood of nodal involvement, the nomogram showed a false-negative rate of 0.04 (95% CI: 0.00, 0.19) in the training set and 0.00 (95% CI: 0.00, 0.31) in the validation cohort. Notably, the single false negative case involved a patient with micrometastasis identified during ultrastaging (Table 5).

## 4. Discussion

Assessing lymph node metastasis is a pivotal factor in determining the most suitable therapeutic approach for EC. Given the significant impact of lymphadenectomy on postoperative quality of life and morbidity, there is an urgent need for a cost-effective, easily accessible, and widely applicable method to evaluate the risk of lymph node metastasis. Our findings have facilitated the development of a four-variable nomogram, capable of accurately predicting the preoperative risk of lymph node metastasis in EC patients.

The selection of myometrial invasion as an ultrasound parameter finds support in the existing literature. In Liro et al.’s study, myometrial invasion ≥50%, measured using TV-US, demonstrated an accuracy of 0.707 (AUC 0.684, 95% CI 0.568–0.801) in predicting lymph node involvement [47]. In our cohort, patients with myometrial invasion ≥50% presented an OR of 3.44 (95% CI 0.61–19.3) for positive lymph nodes. Both TV-US and MRI have demonstrated comparable performance, particularly in the hands of skilled practitioners, for the preoperative staging of EC [25,48].

The distance between the tumor margin and uterine sierosa emerges as an independent prognostic factor for both progression-free survival and overall survival, as highlighted in Pergialiotis et al.’s meta-analysis [49]. Additionally, Chattopadhyay et al. observed that the tumor-free distance, measured on the surgical specimen, is an independent predictor of lymph node involvement (OR 0.74; 95% CI 0.56–0.96; *p* = 0.03) in 288 women with EEC [50]. Estimating the myometrial free margin, via TV-US, demonstrates the capability of predicting lymph node positivity with an accuracy and negative predictive value of 63.8% and 89.02%, respectively (OR 0.842, 95% CI 0.736–0.963), as shown in Liro’s study [51]. In our case series, the MFM shows an OR of 0.73 (95% CI 0.51–1.01)

Eriksson et al. conducted a comparative analysis of the assessment of myometrial and cervical stromal invasion in EC patients, among ultrasound experts and gynecologists. The study revealed that ultrasound experts exhibited higher sensitivity, specificity, and concordance with histopathology in assessing cervical stromal invasion (42%, 95% CI: 31–53%, vs. 57%, 95% CI: 45–68%, *p* = 0.01), while no significant difference was observed for deep myometrial invasion (73%, 95% CI: 66–79%, vs. 73%, 95% CI: 66–79%, *p* = 1.0) [52]. These findings suggest that ultrasound experts and gynecologists performed similarly in predicting deep myometrial invasion.

Tumor size is also known as a predictor factor of lymph node positivity. In Mariani et al.’s study involving 328 patients, low-grade EC with a maximum diameter of less than 2 cm had no positive lymph nodes, and did not benefit from adjuvant radiotherapy. This consideration guided clinicians for years in deciding whether or not to perform pelvic lymphadenectomy before the SLN era [53]. In a multicentric study by Capozzi et al., a tumor diameter > 25 mm was identified as a risk factor for lymph node metastasis, along with the degree of myometrial infiltration, preoperative grading, and CA 125 > 35 U/mL. The authors combined these parameters to create a preoperative scoring system with an AUC of 0.75 and a notably high negative predictive value (94.5%) [34]. However, the paper does not specify the methodology used for the preoperative assessment size of the neoplasm and whether this was consistent across all participating centers. In our study, all patients were evaluated by gynecological ultrasound performed by an experienced operator. We did not observe an association between the anteroposterior diameter of the lesion (when measurable) or endometrial thickness and an elevated risk of positive lymph nodes.

Regarding laboratory parameters, in our cases we observed a correlation between a CA 125 value > 35 U/mL and lymph node positivity, with an odds ratio of 7.74 (95% CI 1.86–32.2). This aligns with findings from Lu et al.’s study, reporting a similar association between CA 125 > 35 U/mL and lymph node metastasis, with an odds ratio of 6.865 (95% CI, 2.481–20.840) [33]. Apart from CA 125, another laboratory parameter correlating with our observed outcomes is the preoperative platelet count. Recent literature reflects a keen interest in the ability of platelets to interact with tumor cells, and their role in metastasis [54,55]. Platelet count, either independently [56,57] or in combination with other laboratory parameters, has emerged as a predictive marker for disease spread at diagnosis and for outcome in oncology [58], particularly among patients with gynecological malignancies [59].

A study conducted by the GOG-Israel group revealed that, among 1482 patients, those with thrombocytosis (platelet count > 400 × 109/L) had a higher probability of being diagnosed with a high-grade neoplasm at an advanced stage, with lymphovascular space invasion (LVSI) and lymph node involvement [36].

When comparing our nomogram with others in the literature, the one proposed by Wang et al. shows similarly high accuracy (AUC 0.916, 95% CI: 0.882–0.949). In particular, the authors emphasize that, among the parameters considered, histological factors such as LVSI and parametrial invasion contribute most significantly to the area under the curve (AUC) [60]. In our statistical analysis, lymphovascular space invasion (LVSI), assessed on the final operative specimen, correlates with lymph node metastasis (*p*-value < 0.001). This finding is not unexpected, as LVSI is one of the most critical risk factors for recurrence, and plays a crucial role in determining adjuvant therapy [21]. Notably, our nomogram bases its foundation solely on preoperative collected information, excluding histological parameters derived from frozen sections or definitive specimen analysis. This decision aimed to reflect real-world scenarios, in which a clinician evaluates patients to determine the appropriate therapeutic approach. Bendifallah et al.’s nomogram also primarily relies on histological parameters like myometrial invasion, grading, and histotype, as well as patient age and ethnicity, with an AUC of 0.79 (95% CI, 0.78–0.80). However, in our sample, age did not show a statistically significant correlation with the probability of lymph node positivity. Although tumor-grading data from diagnostic biopsies were available, we opted to exclude them from our nomogram, due to observed discrepancies between preoperative and definitive histological grading, particularly within the obese population, as previously demonstrated in the case series by Capozzi et al., with a discrepancy rate of 37.7% [61]. In contrast to Bendifallah’s approach, Kang et al.’s study focused solely on preoperative parameters (CA 125 > 35 U/mL, myometrial invasion, suspected extrauterine disease, endometrioid histotype, and suspected lymphadenopathy on imaging) [22]. However, unlike our methodology, they utilized MRI to assess the degree of myometrial infiltration and identify suspected lymphadenopathy. Our decision to rely exclusively on ultrasound parameters stems from the aim of developing a rapid and cost-effective model that can be replicated in different clinical settings. This strategy aligns with the premise shared by Eriksson et al., who conducted a prospective analysis of preoperative anamnestic and ultrasound data from 691 surgically treated patients [27]. In addition to the patient’s age, their model also considered the duration of symptoms. While accurate, their formula for determining the probability of lymph node positivity may be more complex to apply without computer software, compared to utilizing a traditional paper nomogram.

Despite the inclusion of molecular analysis in our patient cohort, the molecular class did not emerge as a significant parameter, and thus was not integrated into the nomogram. These findings might be attributed to the fact that 44% of our patients were categorized in the NSMP class. Each molecular class inherently carries a distinct risk of lymph node positivity, as shown in Jamieson et al.’s study: p53abn in 44.8% of cases, POLEmut in 14.2%, MMRd in 14.9%, and the NSMP class in 10.8%. The study’s conclusion suggests that acquiring molecular data from the preoperative biopsy could introduce a new paradigm for patient risk assessment [19]. Such an approach could enable tailoring surgical procedures by considering not only “classic” risk factors, but also the patient’s molecular class. However, it is important to note that molecular testing is not yet universally accessible in all healthcare settings, particularly in low-income countries. Therefore, our nomogram remains applicable even in scenarios where molecular data are not available, or for patients classified in the NSMP class. Moreover, the molecular class identified from the biopsy might not precisely reflect the neoplasm post hysterectomy [62].

Our study exhibits several strengths. Firstly, all patients underwent lymph node staging and our SLN procedures had a high success rate. In cases where the SLN procedures failed, systematic mono- or bilateral lymphadenectomy was performed in all instances, with the exception of one patient. Secondly, a standardized preoperative work-up was conducted for all study subjects. Experienced ultrasound practitioners carried out all ultrasound examinations following a standardized protocol, ensuring the acquisition of high-quality ultrasound data. Finally, our nomogram is a practical, cost-effective, and user-friendly tool, based solely on preoperative variables, and thus easily applicable to clinical practice. The main limitation of our work lies in its single-center study design, with a relatively modest sample size. Furthermore, although our nomogram has been internally validated, external validation is required to confirm its accuracy. Nevertheless, the risk-prediction nomogram holds the potential to determine which women should undergo lymphadenectomy, based on their individual risk of lymph node metastases, allowing for tailored surgery. It could serve as a preoperative supplement to SLN biopsy, guiding intraoperative decisions on continuing with or refraining from bilateral or side-specific lymphadenectomy in cases of mapping failure or those with high risk of post-operative morbidity. This contributes significantly to planning the expected operation time and the necessity for an experienced surgeon. In 1987, Creasman observed no risk of pelvic and para-aortic lymph node metastases in low-risk EC patients [63]. More recently, Mueller et al. conducted a study on 1044 EC patients undergoing surgery with SLN biopsy, showing that in carcinomas without myometrial invasion and G1/G2 grading, the probability of positive lymph nodes was 0%. In cases of myometrial invasion < 50% and G1 grading, the probability of metastatic lymph nodes was 4%, increasing to 10% with myometrial infiltration > 50% and G1 grading. For G3 tumors without myometrial invasion, the probability of lymph node metastasis is 5%, rising to 24% in cases of infiltration > 50% [16]. These findings demonstrate how systematic lymphadenectomy may result in overtreatment in many cases of SLN failure, aligning with the algorithm proposed by the guidelines [17]. The probability provided by our nomogram can aid in deciding whether to perform systematic lymphadenectomy or not.

## 5. Conclusions

We have crafted a four-variable nomogram, designed to predict the risk of lymph node metastasis in women with EC. This straightforward model’s adaptability renders it suitable for use across diverse clinical settings, aiding gynecological oncologists in preoperative patient evaluations to devise personalized treatment strategies. However, external validation is mandatory for confirming the diagnostic accuracy and effectiveness of this model.

## Figures and Tables

**Table 1:** Patients’ characteristics summary statistics (median and IQR) for continuous variables by Nodal Involvement—Training cohort, N = 210.

Characteristics	All Patients N = 210	Nodal Involvement		*p*-Value ^a^
		No N = 167	Yes N = 43	
**Age at Surgery, years**	60.2 (53.6, 69.4)	60.4 (53.6, 69.4)	58.2 (52.1, 70.4)	0.73
**BMI (kg/m^2^)**	26.5 (23.0, 31.3)	27.0 (23.0, 31.5)	25.7 (22.7, 29.6)	0.66
**CA 125**	18.7 (11.9, 30.6)	16.3 (11.1, 23.4)	41.3 (23.4, 101)	**<0.001**
**Platelet count (10^3^/μL)**	249 (212, 299)	243 (211, 283)	270 (213, 335)	**0.01**
**Lymphocytes (10^3^/μL)**	1.73 (1.40, 2.15)	1.74 (1.43, 2.26)	1.63 (1.32, 2.13)	0.32
**Monocytes (10^3^/μL)**	0.45 (0.36, 0.58)	0.45 (0.36, 0.56)	0.52 (0.35, 0.63)	0.20
**Neutrophils (10^3^/μL)**	4.09 (3.30, 5.22)	3.92 (3.19, 5.04)	4.98 (3.87, 5.68)	**0.002**
**NLR (Neutrophil/Lymphocyte ratio)**	2.31 (1.70, 3.15)	2.24 (1.69, 2.81)	3.13 (1.72, 3.72)	**0.01**
**PLR (Platelet/Lymphocyte ratio)**	142 (113, 188)	138 (112, 178)	169 (117, 231)	0.05
**MLR (Monocyte/Lymphocyte ratio)**	0.26 (0.20, 0.34)	0.25 (0.19, 0.34)	0.29 (0.22, 0.36)	0.08
**Uterine thickness (mm)**	40.0 (33.0, 49.0)	39.0 (33.0, 49.0)	45.0 (36.0, 56.0)	**0.03**
**Endometrial thickness (mm)**	12.8 (7.0, 23.0)	11.0 (6.2, 19.4)	24.3 (14.3, 35.5)	**<0.001**
**Minimum free myometrium (mm)**	4.5 (2.4, 9.0)	5.0 (3.0, 10.4)	1.9 (0.0, 3.3)	**<0.001**
**Lesion diameter (mm)**				
** Sagittal**	29.0 (18.0, 41.0)	25.0 (15.0, 37.0)	42.0 (32.0, 59.0)	**<0.001**
** Antero-posterior**	18.0 (10.0, 27.0)	16.0 (9.0, 24.0)	26.5 (22.0, 41.0)	**<0.001**
** Transverse**	25.0 (16.0, 34.0)	24.0 (14.0, 32.0)	32.5 (25.0, 41.5)	**<0.001**
**d/D (mm)**	0.43 (0.23, 0.64)	0.38 (0.20, 0.60)	0.67 (0.47, 0.78)	**<0.001**
**Distance between tumor and external os (mm)**	24.0 (17.0, 32.0)	26.0 (20.0, 35.0)	17.5 (0.1, 23.5)	**<0.001**
**PCR**	0.20 (0.10, 0.50)	0.20 (0.10, 0.55)	0.25 (0.10, 0.50)	0.84

^a^ Wilcoxon test; IQR = Interquartile Range; d/D = Endometrial thickness/Uterine Antero-posterior diameter ratio.

**Table 2:** Patients’ characteristics summary statistics for categorical variables by Nodal Involvement—Training cohort, N = 210.

Characteristics	Level	Nodal Involvement N (%) ^a^		*p*-Value ^b^
		No	Yes	
**Parity (N = 171)**		107 (78.1)	24 (70.6)	0.37
**Menopausal status**	**Post**	126 (75.5)	33 (76.7)	1.00
	**Pre**	41 (24.6)	10 (23.4)	
**Abnormal Uterine Bleeding**	**None**	59 (35.5)	17 (39.5)	0.97
	**Post-menopausal**	86 (51.5)	22 (51.2)	
	**Heavy menstrual**	5 (3.0)	1 (2.3)	
	**Inter menstrual**	9 (5.4)	1 (2.3)	
	**Unknown**	8 (4.8)	2 (4.7)	
**Hormone Replacement Therapy**	**No HRT**	166 (99.4)	43 (100)	1.00
	**Continuous combined estro-progesterone scheme**	1 (0.6)	0	
**Endometrial echo pattern**	**Non-uniform**	88 (52.7)	14 (32.6)	**0.02**
	**Uniform**	68 (40.7)	22 (51.2)	
	**Unspecified**	11 (6.6)	7 (16.3)	
**Uniform endometrium pattern** **(N = 81)**	**Hyper echogenic**	46 (76.7)	15 (71.4)	0.10
	**Hypo echogenic**	2 (3.3)	3 (14.3)	
	**Iso echogenic**	3 (5.0)	3 (14.3)	
	**Three-layer pattern**	5 (8.3)	0	
	**Unspecified**	4 (6.7)	0	
**Endometrial midline**	**Trilaminar**	84 (50.3)	27 (62.8)	**0.005**
	**Irregular**	53 (31.7)	6 (14.0)	
	**Linear**	10 (6.0)	4 (9.3)	
	**Not linear**	13 (7.8)	0	
	**Unspecified**	7 (4.2)	6 (14.0)	
**Endometrial junction** **(N = 209)**	**Interrupted**	73 (44.0)	29 (67.4)	**0.008**
	**Regular**	42 (25.3)	4 (9.3)	
	**Irregular**	28 (16.9)	3 (7.0)	
	**Undefined**	15 (9.0)	2 (4.7)	
	**Unspecified**	8 (4.8)	5 (11.6)	
**Myometrial invasion (N = 189)**	**No invasion**	51 (34.2)	4 (10.0)	**<0.001**
	**<50%**	63 (42.3)	8 (20.0)	
	**>50%**	25 (16.8)	22 (55.0)	
	**Unspecified**	10 (6.7)	6 (15.0)	
**SLN performed ^c^**		165 (99.4)	20 (47.6)	**<0.001**
**Left SLN Pathology (N = 167)**	**Negative**	145 (98.0)	2 (10.5)	**<0.001**
	**Isolated Tumor Cells**	2 (1.4)	5 (26.3)	
	**Micrometastasis**	0	7 (36.8)	
	**Macrometastasis**	0	4 (21.1)	
	**SLND not found in the specimen**	1 (0.7)	0	
	**Positive, of unknown type**	0	1 (5.3)	
**Right SLN Pathology (N = 185)**	**Negative**	154 (93.9)	4 (19.1)	**<0.001**
	**Isolated Tumor Cells**	8 (4.9)	3 (14.3)	
	**Micrometastasis**	0	7 (33.3)	
	**Macrometastasis**	0	6 (28.6)	
	**SLND not found in the specimen**	2 (1.2)	0	
	**Positive, of unknown type**	0	1 (4.8)	
**Measurable endometrium thickness**		154 (92.2)	41 (95.4)	0.74
**Indentifiable lesion**		131 (78.4)	38 (88.4)	0.20
**Cervical invasion**		10 (6.0)	15 (34.9)	**<0.001**
**Synchronous ovarian cancer**		0	0	-
**LVSI (N = 209)**	**No**	147 (88.6)	21 (48.8)	**<0.001**
	**Yes**	19 (11.5)	22 (51.2)	
**LVSI Type (N = 41)**	**Diffuse**	8 (42.1)	13 (59.1)	0.35
	**Local**	11 (57.9)	9 (40.9)	
**CA 125**	**≤35**	149 (89.2)	21 (48.8)	**<0.001**
	**>35**	18 (10.8)	22 (51.2)	

^a^ Column percent on non-missing values; ^b^ Fisher’s exact test; ^c^ Failed on N = 1 (0.5%) monolateral and N = 1 (0.5%) bilateral; LVSI = Lympho Vascular Space Invasion.

**Table 3:** Nodal involvement univariate odds-ratio estimates for continuous variables—Training cohort, N = 210.

Risk Factor	Level	OR (95% CI)	*p*-Value	AUC (95% CI)
**Age at Surgery, years**		0.94 ^a^ (0.83, 1.06)	0.30	0.54 (0.46, 0.63)
**BMI (kg/m^2^)**		0.81 ^a^ (0.65, 1.02)	0.07	0.56 (0.49, 0.64)
**CA 125**		1.84 ^b^ (1.42, 2.39)	**<0.001**	0.80 (0.73, 0.86)
**Platelet count (10^3^/μL)**		1.27 ^c^ (1.06, 1.54)	**0.01**	0.60 (0.52, 0.69)
**Lymphocytes (10^3^/μL)**		4.08 ^c^ (0.37, 44.8)	0.25	0.45 (0.37, 0.53)
**Monocytes (10^3^/μL)**		NE	0.16	0.56 (0.47, 0.64)
**Neutrophils (10^3^/μL)**		NE	**0.01**	0.60 (0.52, 0.69)
**NLR (Neutrophil/Lymphocyte ratio)**		1.22 ^d^ (0.98, 1.51)	0.07	0.60 (0.51, 0.69)
**PLR (Platelet/Lymphocyte ratio)**		1.00 ^d^ (0.99, 1.01)	0.09	0.59 (0.50, 0.67)
**MLR (Monocyte/Lymphocyte ratio)**		1.70 ^c^ (0.35, 8.26)	0.51	0.58 (0.33, 0.47)
**Uterine thickness (mm)**		1.16 ^a^ (1.06, 1.27)	**0.002**	0.63 (0.56, 0.71)
**Endometrial thickness (mm)**		1.26 ^a^ (1.14, 1.40)	**<0.001**	0.68 (0.59, 0.77)
**Minimum free myometrium (mm)**		0.18 ^a^ (0.08, 0.40)	**<0.001**	0.78 (0.70, 0.86)
**Lesion diameter (mm)**	**Sagittal**	1.25 ^a^ (1.15, 1.36)	**<0.001**	0.74 (0.66, 0.81)
	**Antero-posterior**	1.36 ^a^ (1.21, 1.53)	**<0.001**	0.75 (0.68, 0.82)
	**Transverse**	1.26 ^a^ (1.13, 1.40)	**<0.001**	0.71 (0.63, 0.78)
**d/D (mm)**		31.9 ^d^ (8.04, 127)	**<0.001**	0.72 (0.64, 0.80)
**Distance between tumor and external os (mm)**		0.65 ^a^ (0.55, 0.77)	**<0.001**	0.75 (0.66, 0.84)
**PCR**		1.22 ^c^ (0.91, 1.65)	0.18	0.46 (0.37, 0.56)

^a^ by 5 units increase; ^b^ by 35 units increase; ^c^ by 50 units increase; ^d^ by 1 unit increase; AUC = Area Under Curve; OR = Odds Ratio; 95% CI: 95% Confidence Interval; NE = Not Estimable.

**Table 4:** Best multivariable logistic-regression-analysis model.

Risk Factor Entered	Level	OR (95% CI)	*p*-Value
**CA 125**	**≤35**	ref	**<0.001**
	**>35**	7.74 (1.86, 32.2)	
**d/D**	**<0.50**	ref	**<0.001**
	**≥0.50**	3.44 (0.61, 19.3)	
**Minimum free myometrium**		0.73 (0.53, 1.01)	0.05
**Platelets**		1.01 (1.00, 1.02)	0.13

d/D = Endometrial thickness/Uterine Antero-posterior diameter ratio. Hosmer–Lemeshow Goodness of fit, *p* = 0.22; OR = Odds Ratio; 95% CI: 95% Confidence Interval; ref = reference level.

**Table 5:** Predicted vs. Observed Nodal involvement in the training and validation cohorts at the estimated 10% cutoff probability involvement.

Cohort	Observed N (%) ^a^		
	Predicted	No	Yes
**Training ^b^**	**No**	60 (58.8)	1 (3.7)
	**Yes**	42 (41.2)	26 (96.3)
**Validation ^c^**	**No**	25 (64.1)	0
	**Yes**	14 (35.9)	10 (100)

^a^ Column percent on non-missing values; ^b^ False Positive Rate: 0.41, 95% CI: (0.32, 0.51); False Negative Rate: 0.04, 95% CI: (0.00, 0.19); ^c^ False Positive Rate: 0.36, 95% CI: (0.21, 0.53); False Negative Rate: 0.00, 95% CI: (0.00, 0.31).

## Data Availability

The data presented in this study are available on request from the corresponding author. The data are not publicly available due to patients’ privacy restrictions. The data are safely stored in a private database of the European Institute of Oncology, Milan, Italy.
